# Virtual reality-based balance training system augmented with operant conditioning paradigm

**DOI:** 10.1186/s12938-019-0709-3

**Published:** 2019-08-28

**Authors:** Deepesh Kumar, Nirvik Sinha, Anirban Dutta, Uttama Lahiri

**Affiliations:** 1 Indian Institute of Technology Gandhinagar, Gandhinagar, India; 2 National University of Singapore, The N.1 Institute for Health, 28 Medical Dr., Singapore, 117456 Singapore; 3 Indian Institute of Technology Kharagpur, Kharagpur, India; 4 University at Buffalo, Buffalo, NY USA

**Keywords:** Balance rehabilitation, Center of pressure, Operant conditioning, Stroke, Virtual reality, Wii Balance Board

## Abstract

**Background:**

Stroke-related sensory and motor deficits often steal away the independent mobility and balance from stroke survivors. Often, this compels the stroke survivors to rely heavily on their non-paretic leg during weight shifting to execute activities of daily living (ADL), with reduced usage of the paretic leg. Increased reliance on non-paretic leg often leads to learned nonuse of the paretic leg. Therefore, it is necessary to measure the contribution of individual legs toward one’s overall balance. In turn, techniques can be developed to condition the usage of both the legs during one’s balance training, thereby encouraging the hemiplegic patients for increased use of their paretic leg. The aim of this study is to (1) develop a virtual reality (VR)-based balance training platform that can estimate the contribution of each leg during VR-based weight-shifting tasks in an individualized manner and (2) understand the implication of operant conditioning paradigm during balance training on the overall balance of hemiplegic stroke patients.

**Result:**

Twenty-nine hemiplegic patients participated in a single session of VR-based balance training. The participants maneuvered virtual objects in the virtual environment using two Wii Balance Boards that measured displacement in the center of pressure (CoP) due to each leg when one performed weight-shifting tasks. For operant conditioning, the weight distribution across both the legs was conditioned (during normal trial) to reward participants for increased usage of the paretic leg during the weight-shifting task. The participants were offered multiple levels of normal trials with intermediate catch trial (with equal weight distribution between both legs) in an individualized manner. The effect of operant conditioning during the normal trials was measured in the following catch trials. The participants showed significantly improved performance in the final catch trial compared to their initial catch trial task. Also, the enhancement in CoP displacement of the paretic leg was significant in the final catch trial compared to the initial catch trial.

**Conclusion:**

The developed system was able to encourage participants for improved usage of their paretic leg during weight-shifting tasks. Such an approach has the potential to address the issue of learned nonuse of the paretic leg in stroke patients.

## Introduction

Stroke is a leading cause of disability worldwide [1]. Following a stroke, one’s balance might be impaired that in turn can adversely affect one’s ability to perform activities of daily living [2]. Often, the post-stroke balance impairment is associated with reduced postural control [3] in hemiplegic patients. This is because stroke-related sensory and motor deficits cause hemiplegic stroke patients to rely heavily on their non-paretic leg with reduced usage of the paretic leg for postural adjustment [4]. For the chronic stroke patients, research studies report that the impaired balance may be related to a learned nonuse of the paretic leg (in addition to the paresis caused by stroke) despite probable improvement in motor functionality of the lower limb [5]. The balance impairment due to learned nonuse of the paretic leg can be addressed by encouraging the patients for increased usage of the paretic leg while performing tasks of daily living. In the past, it has been shown that after unilateral forelimb deafferentation, monkeys do not use the affected limb in free situations. However, the same monkey can use the deafferented limb after special training based on operant conditioning approach [6] or while the intact limb is restrained [7]. The operant conditioning approach aims to modify one’s behavior while responding to a task that can be facilitated through the use of reinforcement such as used in studies with patients having spinal cord injury [8]. Originally developed by Skinner et al. [9], it is a learning paradigm centered on the modification of one’s response behavior. This technique has the potential to modify human motor behavior through an appropriately employed reinforcement schedule [10]. Operant conditioning techniques have already been employed in some of the commercial video games [11]. The other variation is constraint-induced movement technique (CIMT). Based on the findings from the deafferented primate research, Taub et al. [12] and Ostendorf and Wolf [13] developed CIMT for upper-limb rehabilitation in stroke patients. The idea of CIMT is to constrain the non-paretic limb and ask the patient to practice tasks using the paretic limb. This technique has been widely used for upper-limb rehabilitation among post-stroke hemiplegic patients. For example, a recent clinical trial has found home-based CIMT to be more effective in improving the perceived use of the paretic arm as compared to conventional physical therapy [14]. However, the use of CIMT for lower-limb rehabilitation has a practical limitation due to the bilateral nature of the balance tasks where constraining one of the lower limbs might offer limitations. Therefore, conventional physical therapy often tries to improve postural stability by using voluntary or compelled weight-shifting tasks toward paretic leg [5]. Over the last 20 years, the body center of pressure (CoP) has been used as an indicator of postural stability during standing weight-shifting activities [15]. Researchers often use force platforms to measure one’s postural stability in terms of displacement in the body CoP during weight shifting [16]. Due to asymmetric body weight distribution across both the legs during standing, the stroke patients show increased postural sway [4] and asynchronous CoP trajectories between both the paretic and non-paretic legs [17]. The body postural sway can be quantified in terms of CoP excursions when participants shift  their body weight around their base of support (BoS) [10] while performing standing balance tasks. Though conventional therapies are promising, these studies assume that the non-paretic leg has a minimum or no deficit and it can compensate for the impairment of the paretic leg while performing the balance task [18]. This assumption may vary for individuals based on the type and location of brain lesion due to stroke. Researchers such as Kim et al. [19] and Parvataneni et al. [20] suggested that, post-stroke, the performance of non-paretic leg differs from that of paretic leg and the difference usually drives to compensate the abilities of the paretic leg. Such compensation mechanism by non-paretic leg might help stroke patient in attaining improved postural stability despite having poor weight-bearing ability on the paretic leg. However, these compensations restrict us to understand the true effect of balance training intervention on the paretic leg. For instance, Kautz and Patten [21] and Kautz et al. [22] showed that even if the non-paretic leg is used just for compensation, the altered sensorimotor state of the non-paretic leg may interfere with the expression of a normal motor pattern with the paretic leg.

Considering the bilateral nature of the balance training task and possible compensation mechanism by the non-paretic leg, it is imperative to develop a mechanism to understand the contribution of the individual leg while a hemiplegic participant performs the balance task. This will not only be useful in assessing the actual improvement in the ability of the paretic leg to bear weight but also help in designing studies that focus on the increased usage of the paretic leg for improving the postural stability. Having access to individual leg’s contribution toward overall balance, one can easily manipulate the contribution of the individual leg in the exercise tasks for increased usage of the paretic leg using implicit reward and punishment mechanism (i.e., the principle of operant conditioning). Similar to CIMT approach, the operant conditioning approach also emphasizes on overcoming the learned nonuse of the paretic limb. However, unlike CIMT, the operant conditioning approach does not require one to constrain the non-paretic limb. Instead, it relies on observing and modifying explicit behaviors using the antecedents (the surrounding environmental factors leading to the behavior) and consequences (the outcome of the behavior in terms of effects on that environment) [23]. This approach has been used in the case of patients with spinal cord injury [8] in the context of locomotion. However, the applicability of operant conditioning to lower-limb-related standing balance has been largely under-addressed.

In the present study, we aimed to understand the implication of operant conditioning on one’s weight-shifting capability during a standing balance training task. For this, we needed to know the contribution of each leg to the overall balance while participants interacted with computer-based balance tasks. There are few studies in the literature where researchers have leveraged the weight-bearing ability of each leg toward balance rehabilitation. For example, Kennedy et al. [24] have used two Wii Balance Board (WiiBB) (for measuring the weight-bearing capacity of each of the two legs of an individual) for a pilot study on balance training with stroke patients and reported positive reaction from both participants and therapists. Again, Ding et al. [25] have used two WiiBBs to develop games to improve balance control in stroke survivors using modified CIMT approach. Though these studies have used separate WiiBB to measure the contribution of each of the two legs toward one’s overall balance, these did not incorporate operant conditioning in their task paradigm. Also, the existing studies have used off-the-shelf games that were mostly designed from an entertainment perspective rather than the balance rehabilitation perspective. Instead of using off-the-shelf games, we designed and offered virtual reality (VR)-based goal-oriented tasks targeted to increase the usage of one’s paretic leg in an implicit and subtle manner through operant conditioning. We chose VR since this can offer the flexibility of task design, controllable challenge levels and individualized feedback [26, 27] while designing the goal-oriented tasks. Our VR-based two WiiBB-assisted balance training system (V2BaT henceforth) used two WiiBBs to measure the CoP of each leg while one stood on the two WiiBBs. While a participant was asked to shift weight, the CoP excursion due to each leg was used as a measure of one’s weight-bearing capability. Based on the observed weight-bearing capability, we used operant conditioning approach to condition the weight-bearing ability of each leg in a goal-oriented VR-based weight-shifting task. Also, among the three balance strategies used by humans [28], the Ankle strategy is the one that is used during the standing balance task. When we stand, our ankle joint allows us to move in all directions and therefore Ankle strategy is the most important tool to maintain our balance while we shift our weight in a different direction [29]. To follow the Ankle strategy, the heel of the user should be in contact with the platform on which the user is standing and performing the weight-shifting tasks. Therefore, in this study, we developed a heel detection module to make sure that participants should follow the Ankle strategy during the balance training session.

The objectives of our present study were twofold, namely to (i) design V2BaT system that can offer VR-based standing balance tasks and (ii) carry out a study to understand the implication of operant conditioning on one’s (a) task performance and (b) ability to increase the usage of the paretic leg.

## Results

In this study, twenty-nine hemiplegic participants were exposed to V2BaT system for a single session of balance training. The V2BaT system used two WiiBBs, one for each leg, to measure CoP displacement for each leg. The weighted sum of CoP for left and right legs was used to maneuver the virtual object (VR_Obj_) in the VR environments ("Materials and method" section). The operant conditioning paradigm was employed during the normal trial (NT) of VR-based balance tasks, where weightage of CoP due to the individual leg was conditioned toward overall CoP displacement. In each difficulty level of normal trial, the weightage of the CoP due to each leg was decided based on the participant’s residual ability to displace his/her overall CoP from the respective baseline position in the anterior direction ("Weight distribution and threshold estimator module" section). The effect of operant conditioning that was applied during the normal trial was captured in the catch trial (CT), where both the legs (paretic and non-paretic) were given equal weightage toward overall CoP displacement. The V2BaT system provided balance tasks of different difficulty levels that were individualized to participants’ residual balance ability and their performance in the VR-based balance tasks. Therefore, the participants were offered normal trials of varying difficulty levels (NT_Level_in_) with intermediate CT tasks ("Task switching rationale" section). In the following subsections, we present our observation on the participants’ improvement in balance in terms of performance score in the VR-based tasks, improved CoP displacement and improved stability in CoP excursion.

### Effect of V2BaT on participants’ performance score

**Fig. 1:**
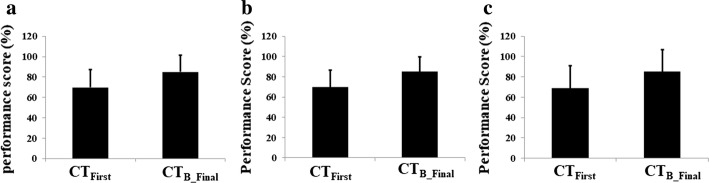
Group average of participants’ performance score (%) for **a** all the participants, **b** left hemiplegic group, **c** right hemiplegic group

Figure 1a shows the group average of the participants’ percentage performance score (%P_f_Score_) in their first catch trial (CT_First_) and best of the final catch trial (CT_B_Final_). A performance score of 70% was considered as an ‘Adequate’ score in the VR-based balance task offered to the participants ("Performance score evaluation module" section). We can see that the participants achieved marginally ‘Inadequate’ performance score on an average in their CT_First_ trial. Though the mean %P_f_Score_ during CT_First_ was found to be close to the threshold for ‘Adequate’ performance, a detailed look at the participants’ performance data indicated that about 50% of the participants had %P_f_Score_ well below the threshold (70%) during CT_First_. In contrast, the mean %P_f_Score_ during CT_B_Final_ was well above the threshold with approximately 22.04% improvement in group average of %P_f_Score_ during CT_B_Final_ as compared to the CT_First_. Almost 70% of the participants had a P_f_Score_ > 80% during the CT_B_Final_. The error bar shown in Fig. 1a indicates the standard deviation (STD) in the performance of all the 29 participants. We observed only a slight reduction (~ 8%) in the amount of variation in the participants’ performance in CT_B_Final_ compared to CT_First_. This was expected since the difficulty of balance tasks was adaptive to one’s residual weight-shifting capability, and due to that, the improvement in performance score was also individualized. Therefore, though they demonstrated improved performance in CT_B_Final_ compared to CT_First_, the variation in performance among the participants did not change by a large amount.

Since our participant pool had a mix of left hemiplegic (*n *= *7*) as well as right hemiplegic (*n *= *22*) patients, we segregated the participants into two groups, namely left hemiplegic group (LH_Group_) and right hemiplegic group (RH_Group_). Figure 1b, c shows a comparative estimate of the %P_f_Score_ during CT_First_ and CT_B_Final_ for the LH_Group_ and RH_Group_, respectively. In both of these figures, we can see a similar improvement in the %P_f_Score_ (Δ% = 21.42% and 24.03% for the CT_First_ to CT_B_Final_ for LH_Group_ and RH_Group_, respectively). Also, from the error bar in Fig. 1b, c, we can see that LH_Group_ showed higher variation in the performance compared to RH_Group_ that can be probably due to fewer participants in the LH_Group_ as compared with that for the RH_Group_. However, the trend in the variation of performance in CT_First_ and CT_B_Final_ was similar for both the participant groups (Fig. 1a).

A dependent sample *t* test carried out between all participants’ %P_f_Score_ in their CT_First_ and CT_B_Final_ tasks showed a significant improvement (*p* value < 0.01) in the performance score. Similar was the observation on improvement in performance for both the LH_Group_ and the RH_Group_ (*p* value < 0.01).

To summarize, the V2BaT system with operant conditioning could motivate the post-stroke hemiplegic participants to increase the contribution of their paretic side during the weight-shifting task. This was evident from the overall statistically significant improvement in %P_f_Score_ from the CT_First_ to CT_B_Final_ trials that used equal weight distribution for each leg while participants maneuvered the virtual object.

### Effect of V2BaT on one’s capability to displace CoP

**Fig. 2:**
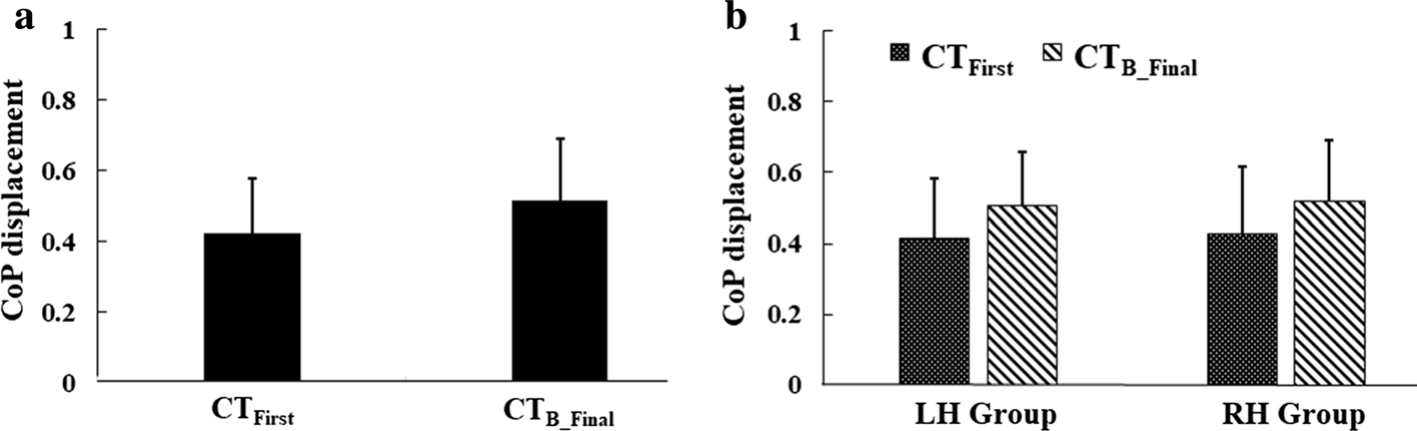
Group average of CoP displacement (normalized) of the paretic leg for **a** all the participants, **b** left hemiplegic and right hemiplegic participants

Figure 2a shows the group average of all participants’ normalized CoP displacement (∆CoP) of the paretic leg while interacting with CT_First_ and CT_B_Final_ tasks. The ∆CoP was normalized with respect to the maximum ∆CoP achieved by the participant pool. We can see that there was an enhancement in the normalized ∆CoP from CT_First_ to CT_B_Final_ for the paretic (Δ% = 21.29%) leg. Again, for the paretic leg, the improvement for LH_Group_ was Δ% = 21.63% and that for the RH_Group_ was Δ% = 21.74% (Fig. 2b, c). A dependent sample *t* test was carried out on the ΔCoP contributed by the participants’ paretic leg from CT_First_ to CT_B_Final_ tasks, and it was found significant (*p* value < 0.01). We also performed a similar test for the LH_Group_ and the RH_Group_. Results showed significant enhancement in terms of ∆CoP (*p* value < 0.01) for both the RH_Group_ and the LH_Group_. Also, the error bars presented in Fig. 2a, b showed that, although there was an overall increment in ΔCoP, the variation in the participants’ ability to displace the CoP remained almost the same while considering CT_First_ and CT_B_Final_ task trials.

**Fig. 3:**
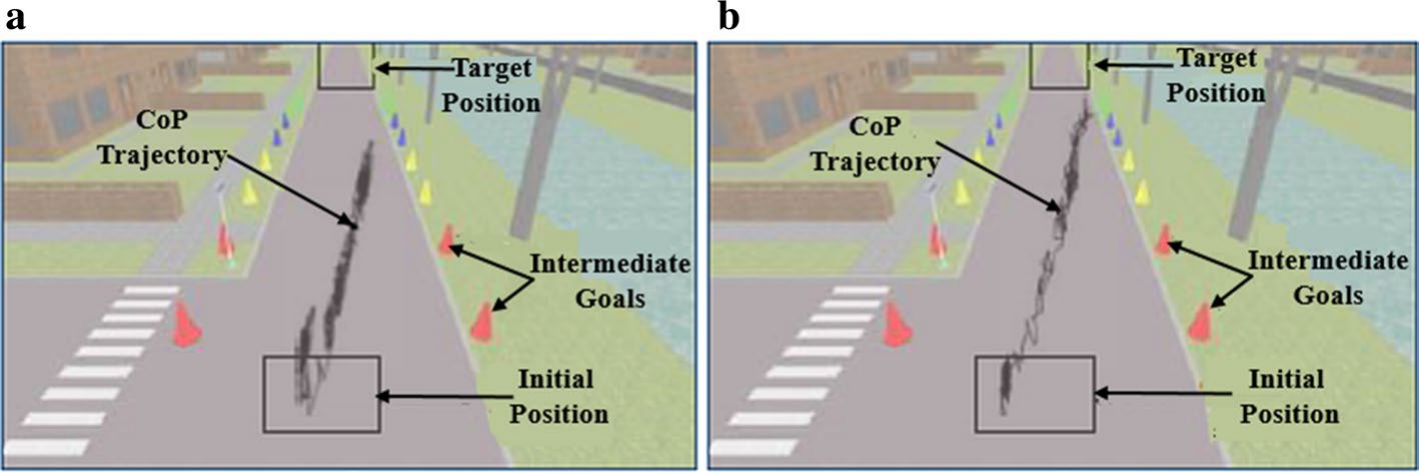
Paretic leg’s CoP trajectory for a typical case in **a** CT_First_, **b** CT_B_Final_; typical case is of participant S23

Further, the quality of weight shifting in a balance task depends not only on the extent of CoP displacement but also on the smoothness of the trajectory of the CoP (stable CoP maneuvering) [30]. Therefore, we wanted to make an in-depth comparative analysis into the trajectory of the CoP due to the paretic leg during CT_First_ and CT_B_Final_ task trials, while a participant maneuvered the virtual object during a weight-shifting task. Here, as a typical case, we chose participant S23 who was right hemiplegic having BBS score of 41 (least among that of the participant pool) (Table 1). Figure 3a, b shows the CoP trajectory of the paretic leg for S23 during CT_First_ and CT_B_Final_ task trials. There was an improvement in terms of greater ΔCoP (%Δ = 21%) in her CT_B_Final_ task trial compared to the CT_First_ task. The improvement in her postural stability can be seen from the enhanced CoP stability as quantified by the CoP trajectory (indicative of reduced postural sway) in the CT_B_Final_ task compared to CT_First_ task. Quantitatively, the spread of CoP trajectory was reduced by 5.86% from CT_First_ to CT_B_Final_ task trials. Thus, we can infer that S23 has not only achieved improved CoP displacement capability (ΔCoP), but also acquired improved control on her postural stability during weight shifting.

**Table 1:** Participants’ clinical characteristics

PI	Age (years)/gender	PS	PSP (years)	BBS	PI	Age (years)/gender	PS	PSP (years)	BBS
S1	26/M	R	1	48	S16	62/M	R	0.33	55
S2	58/M	R	3.5	46	S17	60/M	R	1	51
S3	45/M	L	1	53	S18	53/M	L	0.58	54
S4	43/M	R	0.3	52	S19	55/F	R	4	46
S5	58/M	R	1	46	S20	69/M	L	2	45
S6	52/M	L	5	48	S21	35/M	R	0.58	55
S7	17/M	R	1	54	S22	25/M	R	0.25	53
S8	50/F	R	0.16	NA	S23	63/F	R	2	41
S9	31/M	R	2	53	S24	38/M	R	2	55
S10	55/M	R	1.5	53	S25	55/M	R	0.16	52
S11	54/M	R	0.08	43	S26	45/M	R	7	54
S12	30/M	R	3.5	49	S27	66/M	R	0.16	50
S13	65/M	L	0.08	51	S28	48/M	R	0.16	52
S14	36/M	R	1.5	45	S29	58/M	L	0.02	53
S15	58/M	R	0.16	50					

*PI* participant’s identity, *BBS* Berg Balance Scale, *PS* paretic side, *R* right, *L* left, *PSP* post-stroke period, *M* male, F female

To summarize, we find that the interaction with V2BaT system having operant conditioning contributed to the improvement in terms of the ΔCoP of the paretic leg for both the groups, i.e., RH_Group_ and LH_Group_, along with improved control on one’s postural stability during weight shifting as evident from the CoP trajectory. There was some improvement in terms of enhanced ∆CoP for the non-paretic leg as well that might be due to repetitive exercise with the V2BaT system.

### Effect of V2BaT on participants’ performance during catch trials and normal trials

The V2BaT system offered normal trials (tasks) of varying challenge levels, referred as NT_Level_in_ (*i *= number of trials in a difficulty level and *n *= current difficulty level), using an operant conditioning regime. Also, the catch trials (tasks; *CT*_*i*_; *i *=* 1, 2 …*) were offered in between and at the end of the NT_Level_in_ tasks. The participants were kept unaware of whether the task was a normal trial or a catch trial.

**Fig. 4:**
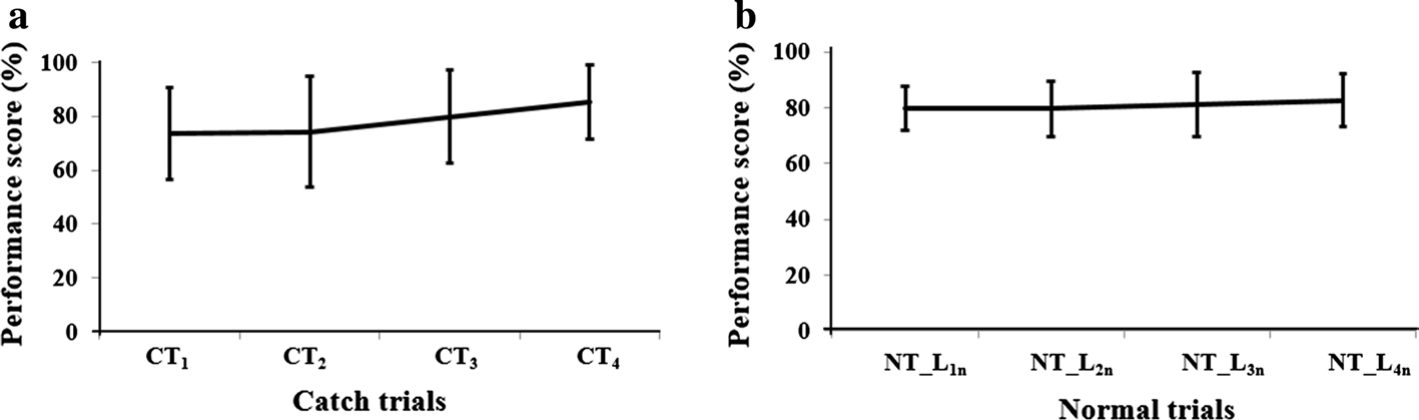
Group average of performance score (%) at each **a** CT, **b** NT; NT_L = NT_Level

Figure 4a, b shows the group average of participants’ %P_f_Score_ at different catch trials and the best score among different task trials within each NT_Level_in_, respectively. For each NT_Level_in_, the number of task trials depended on the individualized performance [following Condition_1_ ("Task switching rationale" section)]. From Fig. 4a, we observe an increasing trend in %P_f_Score_ from CT_First_ to *CT*_*i*_ (where *i* = 2, 3,…). Please note that based on the individualized performance capability, participants were offered different exit points. Specifically, before exiting from interaction with the V2BaT system, each participant was offered a CT_Final_ task trial ("Task switching rationale" section). The idea was to see how a participant performed post the repetitive exercises (offering operant conditioning) in a task in which the weight-bearing capability of one’s paretic leg was considered to be closely similar to that of the non-paretic leg that is indicative of nearly symmetrical body weight distribution. In our participant pool, 82.75%, 62.06% and 27.58% of the participants were able to reach *CTi* with *i *= 2, 3, 4, respectively. Again, from Fig. 4b, we see that the %P_f_Score_ was almost constant across different NT_Level_in_ (based on that achieved by the participant pool) even though every NT_Level_in_ was of increased challenge compared to the previous one as far as the weight distribution was concerned. Since the mean performance scores at different NT_Level_in_ were nearly similar (with small improvement = ~  4 %) across NT_Level_in_ with *i* varying from 1 to 4, we can infer that each NT_Level_in_ with inherent operant conditioning paradigm might have helped the participants to actively compensate the difficulty introduced by the tasks of increased challenge offered by the V2BaT system at least partly through increased usage of the paretic leg.

In short, we can say that even for a limited exposure of one session, we could see the increased usage of the paretic leg by the hemiplegic participants in weight-shifting tasks from what they had when they came in for the study. The improved usage might be a result of the contribution of operant conditioning offered by V2BaT system during NT_Level_in_ through the use of modified weight distribution across both the legs presented in a subtle manner. Also, the gradual improvement in the %P_f_Score_ across the catch trials might be indicative of the residual effect of operant conditioning provided by the NT_Level_in_. We expect that increased exposure to such an environment over a prolonged duration might contribute to further improved performance score representative of enhanced CoP maneuverability across tasks.

## Discussion

Postural instability during the weight-shifting task is associated with patients’ learned nonuse of paretic leg and over-reliance on the non-paretic leg while performing tasks of daily living. The V2BaT system presented in this study was designed to encourage the hemiplegic post-stroke patients to make an increased usage of their paretic leg since it can dramatically improve their overall functional autonomy.

### Use of two balance boards to assess the contribution of each leg toward overall balance

The use of separate WiiBB for each leg allowed us to quantitatively estimate the contribution of each leg of an individual performing the weight-shifting tasks. Taub et al. [5] mentioned that, in addition to the paresis caused by stroke, merely the learned nonuse of the paretic limb might affect the overall functionality. We indeed found that participants who had BBS score more than 50, i.e., almost healthy balance, had significantly reduced CoP maneuvering ability in the paretic leg compared to their non-paretic limb. This indicates that they might have used their non-paretic leg to compensate for the inability of the paretic leg inability during the BBS test, as discussed in [4, 18]. Such an inability would have remained unquantified during the execution of a task unless we measure the ability of each leg of an individual to maneuver the CoP using separate force plate measurement platforms (one for each leg) as used by the V2BaT system. Furthermore, this study is a step forward from the previous studies that used two WiiBBs [24, 25] since we were able to measure and use the CoP displacement data in real time to encourage the increased usage of the paretic leg through the operant conditioning approach.

### Use of virtual reality augmented with operant conditioning

The present study applies the principle of operant conditioning to the lower-limb balance rehabilitation by using the VR-based balance training system. Unlike previous game-based interventions [24, 25] which used ‘off-the-shelf’ commercial games mostly for entertainment rather than rehabilitation, we have designed an interface that can offer the intended intervention while preserving the entertaining and motivational aspects of gaming. For mitigation of boredom and monotonicity, the designed interface offered variations through the use of several task environments, difficulty levels and motivational audio-visual rewards. Again, the use of VR environments with variations was designed to offer motivation to the patients to exercise throughout the balance training. This is important since the literature indicates that motivation is an important factor in rehabilitation and is often linked with improved therapeutic outcomes [31]. In fact, the VR coupled with realistic imagery and sound effects has been shown to offer motivational exercise tasks to the participants [32] undergoing rehabilitation. Again, all this has been done while also keeping in mind not to offer superfluous complexity to the gaming experience that might increase the extraneous cognitive load on the participant. Also, the game engine was programmed in such a way that it provided balance training by adaptively offering tasks of varying challenge levels while tuning to one’s residual weight-shifting capability. Our preliminary results from twenty-nine stroke patients with hemiplegia demonstrated that the V2BaT system augmented with operant conditioning could help in increasing the contribution of their paretic leg toward their overall weight-shifting ability. Questionnaire-based feedback taken at the end of the study from the participants about the usability of the system and their experience while interacting with the VR-based tasks showed that they enjoyed interacting with different templates of the game provided by our system and were interested in future sessions. This indeed aligned with the fact that VR-based rehabilitation system offers increased motivation to the stroke participants [31, 32].

### Use of operant conditioning toward overall balance

An important aspect of operant conditioning is to encourage the participants to make use of their paretic leg by controlling their contribution of each leg toward maneuvering or displacement of their CoP (by using separate weight factors for each leg as in the case of normal trial) while participating in the CoP excursion task set in the VR environment. To uncover the adaptation mechanism that the participants might employ during the normal trial, we exposed them to catch trial in which the weight distribution was made equal for both the legs, without informing them. As can be seen from "Results" section, added to the improvement in their performance score in the V2BaT tasks from CT_First_ to CT_B_Final_, there was also a gradual increment in their performance scores between the subsequent intermediate catch trials. From this observation, we can say that the operant conditioning received by the participants during normal trials (before every intermediate catch trial) might have helped them, at least partially, to improve their directional weight-shifting ability. To understand the contribution of each leg (paretic and non-paretic) toward one’s overall improvement in weight-shifting ability, we analyzed the CoP displacement due to the individual leg during CT_First_ and CT_B_Final_ task trials. While carrying out a comparative analysis between the CoP displacement due to each leg before and post-operant conditioning, our results indicate statistically significant enhancement in the CoP displacement from CT_First_ to CT_B_Final_ for the paretic leg. Additionally, our results indicate a significant enhancement in CoP displacement from CT_First_ to CT_B_Final_ for the non-paretic leg as well. Indeed, there are numerous studies demonstrating ipsilateral sensorimotor deficits following the unilateral stroke [33, 34]. While abnormalities in spinal reflexes might contribute to spread abnormal motor command to the ipsilateral side [35], thereby causing abnormal functionality of the ipsilateral limb (non-paretic side) in hemiplegic stroke patients [36, 37], the improvement in one’s ability in displacing the CoP for the non-paretic leg along with that for the paretic leg might be possibly attributed to the presence of a bilateral component in motor control. Specifically, the bilateral component in motor control is supported by the anatomical evidence that a functional activation might have a bilateral expression in cortical areas [38]. Specifically, in stroke patients, the descending commands while interacting with a network of spinal interneurons and motor neurons might cause an abnormal level of excitability in both sides [39]. This might lead to the hemiplegic stroke patients to suffer from reduced weight-shifting ability in the non-paretic limb as well, thereby leaving the scope of improvement in the weight-shifting ability of the non-paretic leg along with that of the paretic leg. Also, it is likely that the significant improvement in the non-paretic side is due to the transfer of learning from the trained paretic limb as indicated in some of the studies. For example, recently Spampinato [40] has reported the transfer of adaptive visuomotor skill from paretic limb to the non-paretic limb. Indeed, a similar transfer, although in the opposite direction, has been observed by Ausenda et al. [41]. However, while considering adaptive learning, there are two aspects, namely one chiefly concerned with the acquisition of new motor skills and the other being chiefly concerned with the retention of the same [42]. However, it remains an open question (which may be addressed by further longitudinal studies) whether the effect observed in our case is persistent or prone to washout.

### Limitations

Though the preliminary results of V2BaT system are promising, our study had some limitations such as the variability in the duration of the post-stroke period and the laterality of hemiplegia among the participants based on the availability. Also, the participant pool had unequal distribution in terms of the gender of individuals. However, there was no observable difference in the manner in which the female participants (who constituted ~ 10% of the participant pool) executed the V2BaT-based exercises from that of their male counterparts. Thus, we did not segregate the participants based on their gender. Again, the duration of exposure to the system was limited to 1 day only. In the future, we plan to carry out an in-depth longitudinal study by enrolling a larger patient population, categorized based on the extent of residual weight-shifting capability before exposing them to our V2BaT. There is also a possibility of a small degree of uncertainty in the parameters used to evaluate the participants’ performance. For example, we had no way to measure the participant’s engagement level in the task that could have affected his performance.

Similarly, the participant could have put extra effort by adopting an incorrect posture (e.g., hip/knee flexion) which was not compatible with the weight-shifting task used for the present study and can serve as an artifact. Apart from the verbal instruction and careful supervision, we did not have any measures quantifying these artifacts that might have affected the performance. Also, questions remain on the transferability of the skills learned from the VR-based controlled setting to real-life situations. In the future, we plan to carry out longitudinal studies offering multiple exposures to the participants in the balance tasks followed by post-study balance tasks without the VR-based training platform augmented with operant conditioning to understand whether the skills learned in the controlled setting gets translated to real-life tasks.

## Conclusion

The V2BaT system presented in this study could estimate the residual weight-bearing capability of hemiplegic post-stroke participants given a goal-directed balance task. The standing balance task required a participant to displace VR-based virtual objects by maneuvering the CoP through weight shifting while standing on the WiiBB. Accordingly, the V2BaT system conditioned the contribution of both the paretic and non-paretic legs by varying the weight distribution between both the legs (of each patient) in an individualized and subtle manner. The resultant CoP (arising out of the weighted contribution of CoP for each leg) of both the legs was used to maneuver a virtual object in the VR environment. Results of the study indicate the potential of V2BaT system to cause improvement in one’s increased usage of the paretic leg, resulting in improved performance measure while interacting with the VR-based tasks augmented with operant conditioning paradigm. The idea was to encourage the participants to increase the usage of their paretic leg during weight shifting without explicitly directing them to do so and without constraining the abilities of the non-paretic leg. The effect of this implicit conditioning could be seen as increased usage of the paretic leg while maneuvering the virtual object. Specifically, V2BaT system helped the participants to achieve enhanced volitional CoP maneuverability that leads to enhanced CoP stability since external perturbation was volitionally rejected by the participants. However, we need to connect such enhanced volitional CoP maneuverability with changes (or ‘improvement’) in clinical balance scores which is our future work.

## Materials and methods

### Design of V2BaT system

The V2BaT system consisted of (1) VR-based task, (2) weight distribution and threshold estimator, (3) WiiBB–VR handshake, (4) heel lift detection, (5) performance evaluation and (6) task switching modules.

### VR-based task module

In this study, we have designed a database of VR-based standing balance tasks using Vizard software toolkit (from WorldViz LLC.). The database comprised of 72 VR-based tasks in three contextual settings such as land, water and sky. Each setting had variations. For example, land-based settings projected task environments that had skaters on the road, skiers on ice, etc. For water-based setting, there were swimmers under water, etc. For the sky-based setting, there were flying helicopters, birds, etc.

**Fig. 5:**
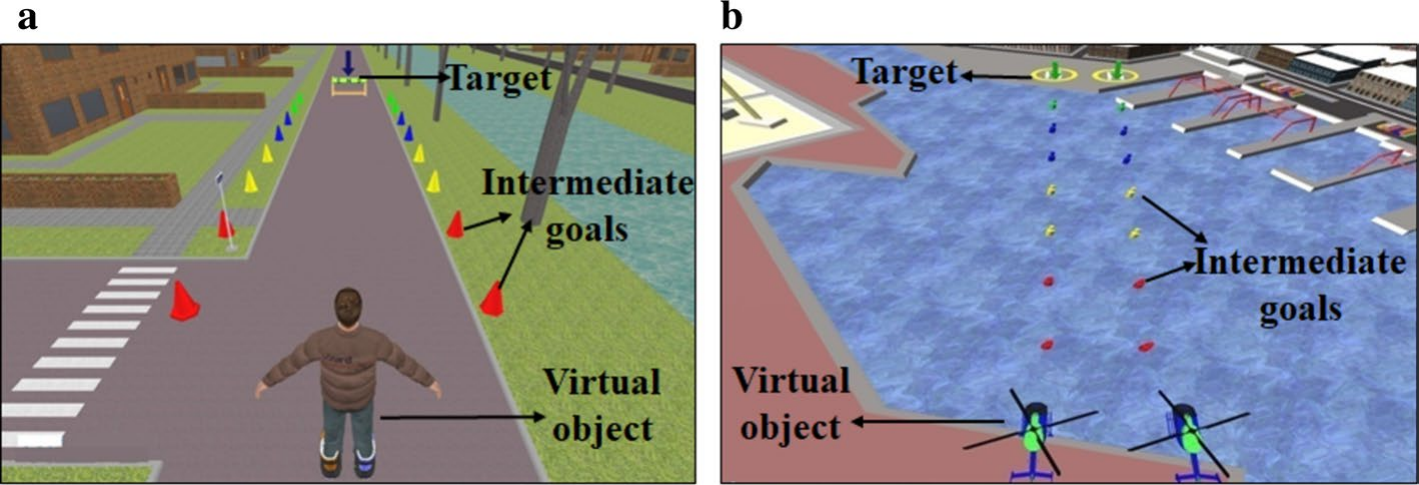
Various templates of VR-based balance rehabilitation tasks of V2BaT system

Figure 5 shows two task environments based on land and water. In each task environment, a context-relevant virtual object, an end goal/target and intermediate milestones were used. For example, in land-based task environments (Fig. 5a), the virtual object was an avatar standing on a road while wearing roller skates on both legs, the end target was a divider at the end of the road, and intermediate milestones were traffic cones located at regular intervals on either side of the road. The end target was meant to be a final milestone to be reached to complete a task. The intermediate milestones were used to help the participant to be able to gauge his/her improvement in CoP maneuvering ability within different tasks. As the participant standing on the two WiiBBs gradually shifted his/her body weight in the anterior direction, the virtual object moved forward toward the end target that was anterior to its initial position in the VR environment (thus reflecting the participant’s weight shifting). As soon as the virtual object crossed the successive intermediate goals (in this case, the traffic cones), these appeared to move backward outside the participant’s view. The idea was to help the participant to gauge his/her ability to shift weight in the anterior direction along with a visual metric in terms of intermediate goals moving backward. In other words, this can offer an individualized metric to monitor the improvement in CoP maneuvering capability in each trial of the tasks. A variation in the degree of task difficulty was also a part of the system design. However, depending on the task difficulty, the scale of displacement of the virtual object with CoP excursion (due to weight shifting) was different for each environment. The degree of task difficulty depended on the conditioned weight distribution (described below in "Weight distribution and threshold estimator module" section) among both the legs of a participant. Here, the tasks were designed to offer visual feedback on one’s directional weight-shifting capability. We chose standing balance tasks that require only the anterior weight-shifting tasks instead of mediolateral weight shifting so that our post-stroke hemiplegic participants do not take advantage of the dominance of one leg compared to the other during shifting weight in the mediolateral direction.

Once a participant completed a task, the V2BaT system provided feedback based on his/her performance. The feedback was given audio-visually through a short audio tone along with one star (*) (akin to the reward system typical of popular commercial computer games). Based on the performance in a task, a participant could receive 1 to 5 stars. Also, V2BaT encouraged the participants by saying either ‘Well done; you are doing great’ (for ‘Adequate’ performance or ‘Keep trying, you can do better’ (for ‘Inadequate’ performance) using pre-recorded audio files.

### Weight distribution and threshold estimator module

**Fig. 6:**
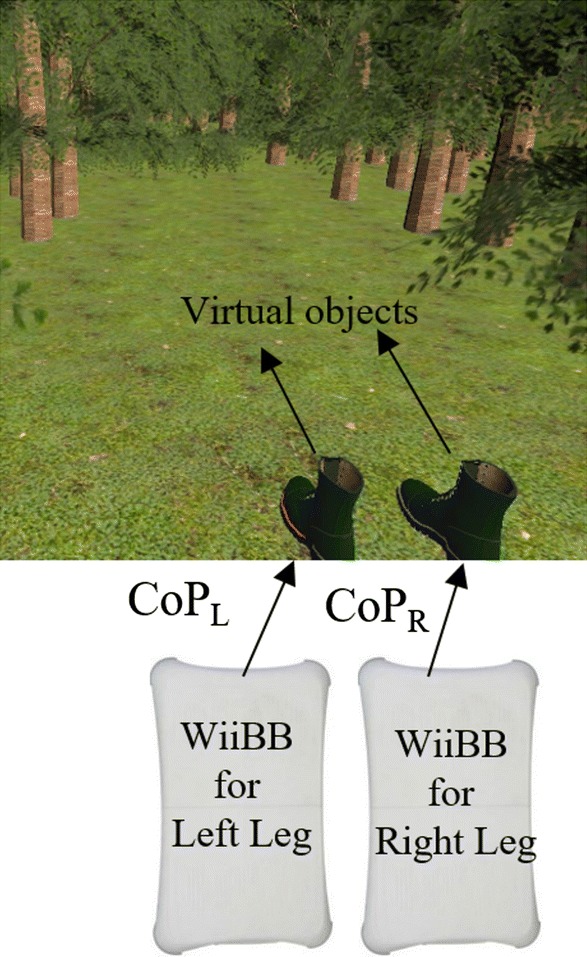
VR-based task designed for the estimation of individualized residual weight-shifting ability

Our participants were hemiplegic with different weight-bearing abilities on either of their legs and with a spectrum of post-stroke balance disorders. Thus, it was important that the conditioned weight distribution across both the legs of a participant was individualized. Before interacting with V2BaT system, we captured one’s individualized residual weight-bearing ability for each leg. We developed a VR-based weight-shifting task (Fig. 6), specifically designed to estimate one’s residual ability to displace the CoP in the anterior direction. This task was used to compute the individualized threshold for CoP displacement that corresponded to a maximum of 100 points (on a 0–100 scale) in a task.

Figure 6 shows the VR-based task that projected a VR world with a pair of boots, one for left and other for right leg placed in a forest environment. This task required the participant to move the boots in the anterior direction as far as possible inside the forest by shifting weight in anterior direction with each boot being controlled by the CoP displacement due to each leg (measured by two WiiBBs). Then, our system computed the range of movement (representing the CoP displacement) of each boot from their initial position (that is while standing upright without weight shifting). Based on a few trials, the maximum CoP displacement *(*∆CoP_max_L_ and ∆CoP_max_R_) was computed for both the right and left legs, respectively. Then, the higher CoP displacement among ∆CoP_max_L_ and ∆CoP_max_R_ was chosen as overall maximum CoP displacement *(*∆CoP_max_). Subsequently, the individualized threshold (∆CoP_THRESH_) was estimated [Eq. (1)]:

1 $$\Delta {\text{CoP}}_{\text{THRESH}} = \, ( 1+ \delta ) \, \Delta {\text{CoP}}_{\rm{max} } .$$

The factor $\delta$ ($\delta$ = 0.2, chosen as an initial approximation and maintained constant throughout the V2BaT-based exercise session) was introduced to intentionally decide the ∆CoP_THRESH_ exceeding the participant’s best possible weight-shifting capability at the beginning of the task (in which the participant was asked to displace the virtual boots as far as possible in the forest environment). This was to accommodate the conservative weight shifting of the post-stroke patients while interacting with the VR-based task developed for estimating ∆CoP_THRESH_. Subsequently, the participants were asked to interact with the tasks offered by V2BaT system. To achieve the conditioned weight distribution while executing the V2BaT task, we needed to estimate one’s initial weight distribution [*W*_L_ini_ and *W*_R_ini_; Eqs. (2) and (3)] as far as left and right legs of hemiplegic participants were concerned followed by updated weight distribution corresponding to each task:

2 $${\text{W}}_{{{\text{L}}_{\text{ini}} }} \left( {\text{\%}} \right) = \left( {\frac{{\Delta {\text{CoP}}_{{{ \rm{max} }\_{\text{L}}}} }}{{\Delta {\text{CoP}}_{{{ \rm{max} }\_{\text{L}}}} + \Delta {\text{CoP}}_{{{ \rm{max} }\_{\text{R}}}} }}} \right) \times 100$$

3 $${\text{W}}_{{{\text{R}}_{\text{ini}} }} \left( {\text{\%}} \right) = \left( {\frac{{\Delta {\text{CoP}}_{{{ \rm{max} }\_{\text{R}}}} }}{{\Delta {\text{CoP}}_{{{ \rm{max} }\_{\text{L}}}} + \Delta {\text{CoP}}_{{{ \rm{max} }\_{\text{R}}}} }}} \right) \times 100 .$$

Here, sign ‘×’ represents the scalar multiplication.

### WiiBB–VR handshake module

During the balance training, the position of the virtual object in the VR environment ("VR-based task module" section) was controlled by the weighted sum of the CoPs obtained from the two WiiBBs. As the V2BaT tasks progressed, we monitored one’s task performance and accordingly we went for updating the weights *W*_L_ and *W*_R_ (with initial weight being *W*_L_ = *W*_L_ini_ and *W*_R_ = *W*_R_ini_) across both the left and right legs. Since the task was to shift one’s weight in the anterior direction, we used only the ‘*y*’ component of the CoP (CoP displacement along the anterior direction) for navigating virtual object for display. However, both ‘*x*’ and ‘*y*’ coordinates of CoP were stored for subsequent offline analysis. The raw CoP data acquired at 30 Hz were processed by a 5-point moving average filter. The position of the virtual object was determined from the filtered CoP data by using Eq. (4):

4 $$\left[ {\text{y}} \right]_{{{\text{VR}}_{\text{OBJ}} }} = {\text{W}}_{\text{L}} \left[ {\text{y}} \right]_{{{\text{CoP}}_{\text{L}} }} + {\text{W}}_{\text{R}} \left[ {\text{y}} \right]_{{{\text{CoP}}_{\text{R}} }}$$

where *W*_L_ and *W*_R_ are the task-specific weight factors; $\left[ y \right]_{{{\text{CoP}}_{\text{L}} }}$ and $\left[ y \right]_{{{\text{CoP}}_{\text{R}} }}$ indicate the ‘*y*’ coordinate of the CoP as measured by the WiiBBs for the left and right leg, respectively.

### Heel lift detection module

**Fig. 7:**
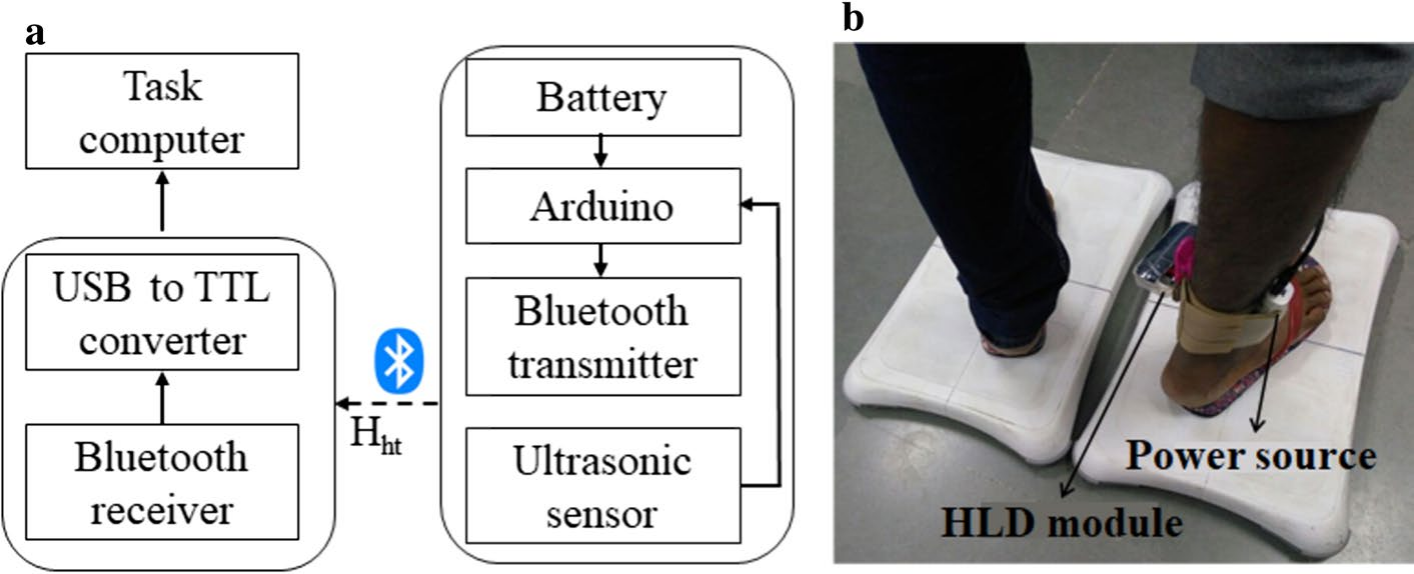
**a** Block diagram and **b** placement of heel lift detection module on the paretic leg of the participant

We wanted to ensure that the participants followed Ankle strategy, an important requirement during standing balance task [43]. Thus, the participants were asked not to lift their heel from the surface of WiiBB while shifting their weight. To identify whether the Ankle strategy was ‘Followed’ or ‘Not Followed,’ we used an ultrasonic sensor-based heel lift detection module (Fig. 7a) that wirelessly communicated the height of the heel above the BoS (surface of WiiBB) at 60 samples/sec to V2BaT system. First, the heel lift detection module was initialized. For this, one was asked to stand upright with his heels touching the surface of WiiBB, and the initial distance (*d*_ini_ (mm)) between the ultrasonic sensor mounted on the participant’s paretic leg (Fig. 7b) and the surface of WiiBB (BoS) was measured. While the participants performed the weight-shifting task, our system continuously measured the distance known as the instantaneous distance (*d*_ins_) between the ultrasonic sensor and BoS. The two distances, i.e., initial distance (*d*_ini_) and instantaneous distance (*d*_ins_), were used to detect one’s heel lift.

The output from the ultrasonic sensor was transmitted wirelessly to the task computer to detect the heel lift via a microcontroller-based circuit. The decision of whether Ankle strategy has been ‘Followed’ or ‘Not Followed’ was taken based on the following equations:

5 $${\text{~Ankle~strategy}} = \left\{ {\begin{array}{*{20}c} {{\text{Followed;}}\quad ~~{\kern 1pt} \quad if~d_{{{\text{ins}}}} < d_{{{\text{Limit}}}} } \\ {{\text{Not~followed}};\quad ~if~d_{{{\text{ins}}}} \ge d_{{{\text{Limit}}}} } \\ \end{array} } \right.$$

6 $$d_{\text{Limit}} = d_{\text{ini}} + d_{\text{th}} ,$$

where *d*_th_ = 20 mm = height tolerance. For details on heel lift detection module, please see our companion paper [44]. If the Ankle strategy was ‘Not Followed,’ then a penalty factor was added to the performance score (described below). Otherwise, no penalty factor was considered.

### Performance score evaluation module

While the participants performed VR-based tasks, the V2BaT system computed their performance scores. The first performance metric P_S1_ [Eq. (7)] looked into the CoP displacement:

7 $${\text{P}}_{{{\text{S}}1}} = 100 - \left( {\frac{{T_{\text{L}} - T_{\text{D}} }}{{T_{\text{L}} }}} \right)100.$$

Here, *T*_L_ = length of the straight path between the initial and end target positions; *T*_D_ = length of one’s CoP displacement (ΔCoP) in the VR environment during weight shifting. The second performance metric P_S2_ [Eq. (8)] was used to penalize the participant for not following Ankle strategy. The penalty was decided from the duration a participant lifted his heel (*T*_Lift_) as a percentage of the total task completion time (*T*_CT_).

8 $${\text{P}}_{{{\text{S}}2}} = \left( {\frac{{T_{\text{Lift}} }}{{T_{\text{CT}} }}} \right)100 .$$

The final percentage performance score (%P_f_Score_) was calculated as

9 $${\text{P}}_{{{\text{f}}\_{\text{Score}}}} = {\text{P}}_{\text{S1}} - {\text{P}}_{\text{S2}} .$$

The V2BaT system was made adaptive to one’s task performance score. One’s performance was considered as ‘Adequate’ or ‘Inadequate’ based on the percentage performance score. For example, if the score was ≥ 70%, then it was considered as ‘Adequate,’ else ‘Inadequate.’ Please note that the threshold of 70% for the performance score was taken as an initial approximation since a performance score of 70% can be considered as satisfactory during initial sessions of exercise in robot-assisted rehabilitation tasks [45], technology-assisted skill learning [46], etc. This can be adjusted based on the study design.

### Task switching module

#### The rationale of operant conditioning

In this study, we used operant conditioning paradigm for balance training through an implicit and subtle cueing technique, presented subliminally by gradual, individualized and controlled variation of the weight distribution across both the legs during the weight-shifting task. This was achieved by subtly increasing the weight contribution [i.e., weightage *W*_L_/*W*_R_ in Eq. (4)] for the paretic leg so that if a participant increased the usage of the paretic leg to displace virtual object, then the V2BaT system rewarded him/her with a higher displacement in virtual object that in turn resulted in higher performance score in the task. For this, the V2BaT was programmed to offer balance tasks of different difficulty levels coupled with the reward based on the task performance that can be considered as a representative of one’s weight-shifting ability.

#### Task switching rationale

**Fig. 8:**
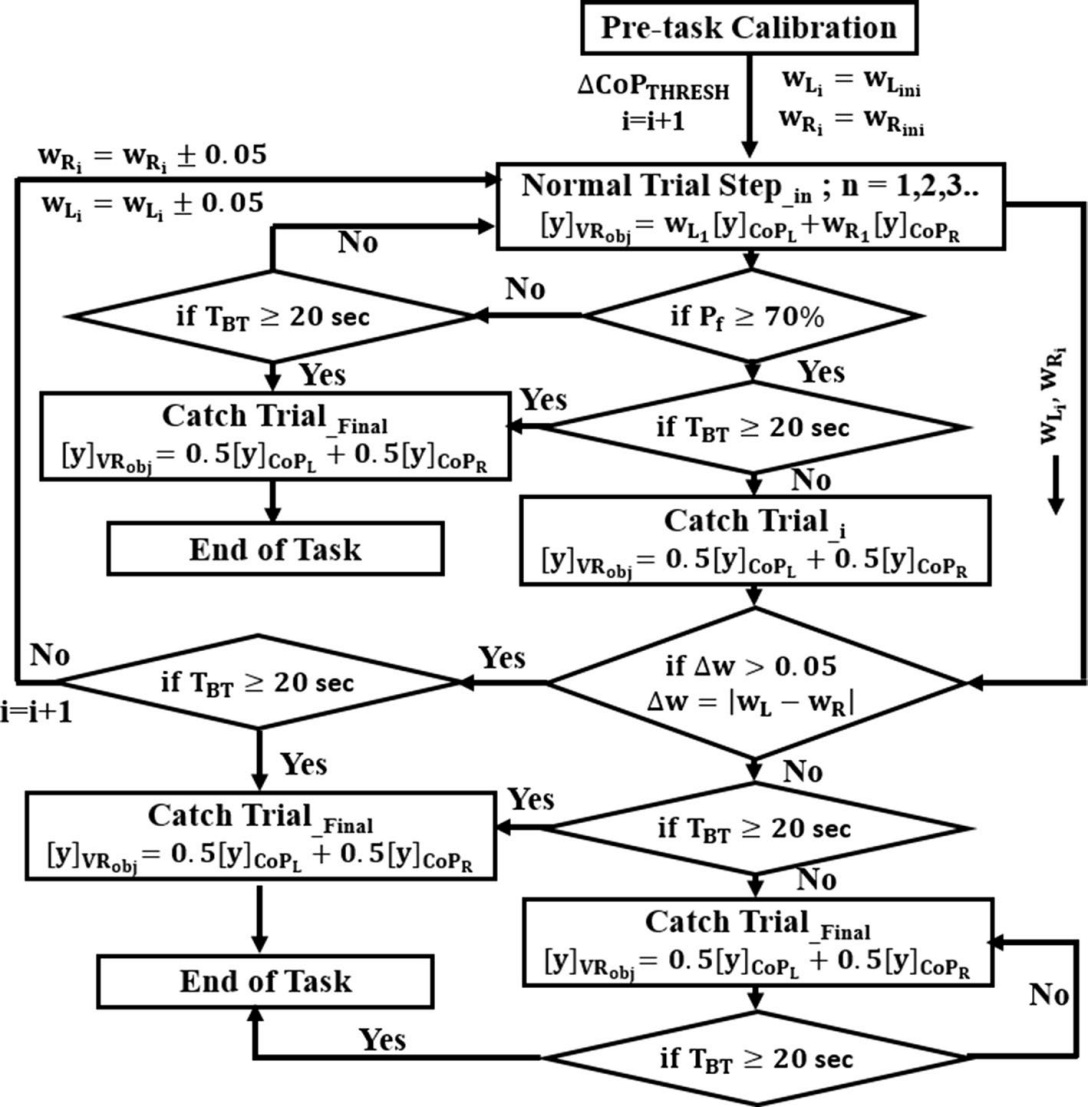
Flow diagram of task switching module of V2BaT system. TBT total time of balance training session, Δ = Δ′ = 0.05

Post the VR-based task offered for estimating $\Delta {\text{CoP}}_{\text{THRESH}}$ ("Weight distribution and threshold estimator module" section), the participants were invited to start interacting with VR-based tasks ("VR-based task module" section) offered by V2BaT using the task switching rationale designed with an implicit operant conditioning regime (Fig. 8). The task switching was done using two conditions, namely Condition_1_ and Condition_2_:

10 $$\begin{aligned} & {\text{Condition}}_{1}{:}\,\% P_{{{\text{f}}\_{\text{score}}}} \ge 70\% \,(`{\text{Adequate'}}) \hfill \\ & {\text{Condition}}_{2}{:}\Delta {\text{W}}\left( { = \left| {W_{\text{L}} - W_{\text{R}} } \right|} \right) > \Delta . \hfill \\ \end{aligned}$$

In Eq. (10), P_f_Score_ is the final percentage performance score [see Eq. (9)] in a task trial. The quantity $\Delta W$ is the absolute difference between task-specific weight factors for the left leg (*W*_L_) and right leg (*W*_R_), respectively. The parameter $\Delta$ is an arbitrary threshold value for the difference between the weightage allotted to one’s left and right legs. The value of $\Delta$ (= 5%) was considered as an initial approximation and can be changed based on the study design.

Here, the tasks were of two types, namely (i) catch trial (*CT*) and (ii) normal trial (*NT*). In the *CT*, equal weightage, i.e., *W*_L_ = *W*_R_ (Eq. (4), similar to the study by JhonBabič [47]) was allocated to each of the paretic and non-paretic legs of hemiplegic post-stroke participants. In *NT*, the weightage allocated to each of the paretic and non-paretic legs was not equal. Specifically, *W*_L_ and *W*_R_ were updated keeping the operant conditioning in mind. The *NT* tasks were of different challenge levels (*NT_Level*) based on the distribution of weights, e.g., values of *W*_L_ and *W*_R_. For a task in the first *NT* challenge level (*NT_Level*_*1*_), the weightage was *W*_L1_ = *W*_L_ini_ and *W*_R1_ = *W*_R_ini_. For subsequent *NT_Level*, the weightage for the paretic and non-paretic legs was increased and decreased by a factor of Δ (5% in this case), respectively. The value of Δ was chosen as an initial approximation, and it can be changed based on the study design. The values of *W*_L_ and *W*_R_ were continuously updated as long as the difference between *W*_L_ and *W*_R_ (i.e., Δ*W* = |*W*_L _− *W*_R_|) was greater than Δ [Condition_2_ in Eq. (10)]. Also, the participants were switched from one *NT_Level* to the next only when they scored ‘Adequately’ in the task belonging to an *NT_Level* (Condition_1_), and Condition_2_ was also satisfied. Else, the participant was offered tasks (i.e., task trials) with the same weightage (i.e., without updating *W*_L_ and *W*_R_) until the participant scored ‘Adequately’ (i.e., P_f_Score_ ≥ 70%). Thus, for a particular *NT_Level,* there could be ‘*n*’ task trials and represented as NT_Level_in_ where ‘*i*’ represents the challenge level. Also, the V2BaT system offered intermediate *CTs* (single-task trial) before switching the challenge level of *NT*. Our idea was to (i) help the participants learn to increasingly use their paretic leg while exercising during *NT* task trials and (ii) help us to understand the effect of operant conditioning on the weight-bearing capability of the paretic leg under real-life situations when one is expected to use both the legs to a similar extent (i.e., *CT* task trial).

The total time of balance training (T_BT_) was 20 min. The task execution started with NT_Level_in_ tasks (*W*_L_ = *W*_L1_ = *W*_L_ini_ and *W*_R_ = *W*_R1_ = *W*_R_ini_), with *i* = 1 and *n* increasing till Condition_1_ was not satisfied or T_BT_ ≤ 20 min. Once the Condition_1_ was satisfied, V2BaT offered *CT*_*i*_ task of the single trial while storing the weightage factors (*W*_L_ and *W*_R_) used in the completed NT_Level_in_ task trial. Note that the *CT*_*i*_ for *i *= 1 was considered as CT_First_ task. Subsequently, we checked for the Condition_2_ (Cases 1 and 2) before going ahead with the next NT_Level_in_ (*i *> 1).

Case_1_: If Condition_2_ was satisfied, then V2BaT system offered a task of next NT_Level_in_ with *i *= 2 to the participant. At this NT_Level_in_ (*i *= 2), the V2BaT system offered several normal trials (*n *= 1, 2, 3…) till the participant’s P_f_Score_ ≥ 70%. Subsequently, next *CT* task, i.e., *CT*_*i*_ with *i *= 2, was offered by the V2BaT system. This whole process was repeated till the Condition_2_ failed or T_BT_ > 20 min.

Case_2_: If the Condition_2_ was not satisfied, then V2BaT system offered *CT* task repetitively until T_BT_ > 20 min. In this case, the offered catch trials were considered as CT_Final_ task trials.

Again, there can be two variations in participation. For example, one variation can be that a participant completed the task execution offered by V2BaT system while staying in Case_1_ until 20 min was over. Then, the V2BaT system terminated the VR-based training by offering the last task as a *CT*_*i*_ task with *i* = Final. The other variation can be that the participant reached Case 2 before completion of 20 min. In that case, the V2BaT system offered several *CT*_*i*_ (with *i* = Final) task trials until 20 min was over. At the end of 20 min, the V2BaT system offered one additional CT_Final_ task (for the sake of similarity to that for Case_1_). Thus, for Case_2_, there were several CT_Final_ tasks. We were interested to understand the best performance of a participant at the end of the VR-based balance training. For the participants concluding the task execution while being in Case_2_, we wanted to understand the best performance achieved by the participant out of the number of CT_Final_ tasks. Instead of considering the performance for the last task of the CT_Final_ tasks, we chose the one among the CT_Final_ tasks for which the participant scored the maximum (i.e., best of final CT task trials [CT_B_Final_, henceforth)] so as to avoid the effect of any monotony arising out of no variation in the challenge level on the performance. However, if a participant remained in Case_1_ till T_BT_ > 20 min, then we had no option rather than considering the performance during the last, i.e., CT_Final_ task (offered just after completion of training duration, i.e., T_BT_ = 20 min) as the CT_B_Final_.

### Participants

The study was carried out in hospital settings after informed consent. Twenty-nine hemiplegic post-stroke survivors (S1–S29) [mean (SD) = 49.55 years (13.89)] volunteered in the study. They had varying residual balance and post-stroke periods (Table 1). The inclusion criteria were (1) ability to follow the instructions and (2) ability to stand and shift weight without orthopedic aids.

### Experimental setup

**Fig. 9:**
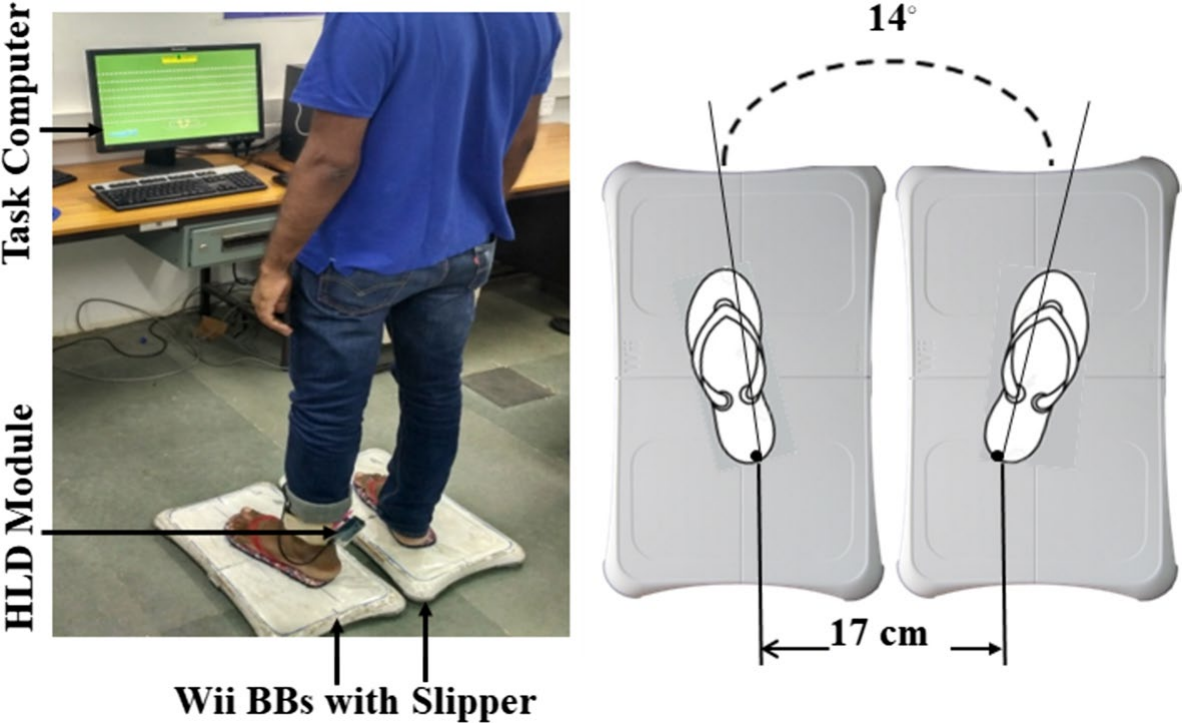
**a** Experimental setup of V2BaT system, **b** foot placement on double WiiBBs for V2BaT system

Figure 9a shows the experimental setup that consisted of (i) two WiiBBs, (ii) a pair of slippers, (iii) a heel lift detection module and (iv) a task computer (PC). The two WiiBBs were placed 1 mm apart on the ground. To avoid fluctuation in the CoP values due to the participant’s movement affecting the computation, the WiiBBs were fitted with slippers. This was necessary as the initial position of the virtual object was calibrated to one’s initial position at the start. The position of the slippers was maintained (Fig. 9b) similar to the setup used by Mansfield et al. [17]. A heel lift detection module was used to monitor whether the Ankle strategy was followed or not followed.

### Procedure

Our study required a commitment of approximately 45 min from each participant. Once a participant arrived in the experiment room, he/she was asked to sit and relax for 5 min. Then, a physiotherapist in our team assessed the participant’s residual balance using the Berg Balance Scale (BBS) [48] measurement and also ensured that the inclusion criteria were satisfied. Subsequently, the experimenter explained the experimental setup and demonstrated the VR-based tasks to the participant. This was followed by the administration of consent form signing by the participant. Additionally, we also told the participant that he/she was free to quit or take breaks in between the balance training session at any time in case of discomfort.

Once the participant was ready, the experimenter fitted the heel lift detection module to the participant’s paretic leg and asked him/her to stand upright with his/her feet in the slippers attached to the WiiBB (Fig. 9a). Then, the experimenter started the study by exposing the participant to VR-based task designed for estimating ∆CoP_THRESH_ ("Weight distribution and threshold estimator module" section). In this task, the experimenter asked the participant to stand upright for 10 s so that their baseline CoP due to left and a right leg can be estimated. Also, we recorded the initial distance (d_ini_) between the ultrasonic sensor of the heel lift detection module and the surface of WiiBB. Followed by this step, the participant was asked to shift weight as much as possible from the initial position in the anterior direction while following the Ankle strategy to displace the virtual objects (pair of the boot in Fig. 6) as far as possible in the forest. This process was repeated three times, and the maximum CoP displacement achieved by individual leg has been used to estimate ∆CoP_THRESH_ as mentioned in "Weight distribution and threshold estimator module" section. Once the threshold CoP displacement was estimated for a participant, he/she was offered the VR-based tasks of different templates (section ‘*VR*-*based Task Module’*) for 20 min following the rules of the game engine described in "Task switching rationale" section.

### Statistical analysis

While the participants interacted with our VR-based tasks during Stage 2, the V2BaT system offered various NTs of various challenge levels and intermediate CTs. Also, it computed their performance, i.e.,  %P_f_Score_ (section ‘*Performance Score Evaluation Module’*) and recorded displacement in CoP (∆CoP) due to the individual leg. We were interested to understand whether the operant conditioning paradigm using V2BaT system contributed to any statistical improvement in one’s performance and enhanced displacement in CoP from their first CT, i.e., CT_First_ task to the best of final CTs (CT_B_Final_) task. The Shapiro–Wilk test of normality on the participants’ performance and ∆CoP data corresponding to CT_First_ and CT_B_Final_ revealed that these were normally distributed. Subsequently, we performed a Student’s t test with a significance level set at *p* value < 0.05 to check the significance of the improvement.

## Data Availability

The datasets used and analyzed during the current study are available from the corresponding author on reasonable request.

## Abbreviations

BoS: base of support
CIMT: constraint-induced movement theory
CoP: center of pressure
ΔCoP_max_: maximum CoP displacement achieved by the participant using either of the legs in the calibration task
ΔCoP_max_: maximum of ∆CoP_max_L_ and ∆CoP_max_R_
∆CoP_max_L_: maximum CoP displacement achieved by the participants using their left leg in the calibration task (before exposing them to balance task augmented with operant conditioning)
∆CoP_max_R_: maximum CoP displacement achieved by the participants using their right leg in the calibration task (before exposing them to balance task augmented with operant conditioning.)
ΔCoP_THRESH_: required CoP displacement to achieve the full score in the VR-based balance task. ΔCoP_THRESH_ = (1 + *δ*)*ΔCoP_max_; *δ* = 0.2
CT: catch trial
LH_Group_: group of left hemiplegic participants
NT: normal trial
P_f_Score_: performance score in the virtual reality-based tasks
RH_Group_: group of right hemiplegic participants
T_BT_: total time of balance training session
V2BaT: virtual reality-based 2 balance board-assisted balance training
VR: virtual reality
WiiBB: Wii Balance Board
